# Identifying Long Non-coding RNA of Prostate Cancer Associated With Radioresponse by Comprehensive Bioinformatics Analysis

**DOI:** 10.3389/fonc.2020.00498

**Published:** 2020-04-07

**Authors:** Meng Xu, Shiqi Gong, Yue Li, Jun Zhou, Junhua Du, Cheng Yang, Mingwei Yang, Fan Zhang, Chaozhao Liang, Zhuting Tong

**Affiliations:** ^1^Department of Radiation Oncology, The First Affiliated Hospital, Anhui Medical University, Hefei, China; ^2^Department of Otolaryngology, Ruijin Hospital, School of Medicine, Shanghai Jiaotong University, Shanghai, China; ^3^Department of Urology, The First Affiliated Hospital, Anhui Medical University, Hefei, China; ^4^Institute of Urology, Anhui Medical University, Heifei, China

**Keywords:** long non-coding RNA, prostate cancer, radioresponse, bioinformatics analysis, WGCNA

## Abstract

Although radiotherapy is greatly successful in the treatment of prostate cancer (PCa), radioresistance is still a major challenge in the treatment. To our knowledge, this study is the first to screen long non-coding RNAs (lncRNAs) associated with radioresponse in PCa by The Cancer Genome Atlas (TCGA). Bioinformatics methods were used to identify the differentially expressed lncRNAs and protein-coding genes (PCGs) between complete response (CR) and non-complete response (non-CR) groups in radiotherapy. Statistical methods were applied to identify the correlation between lncRNAs and radioresponse as well as lncRNAs and PCGs. The correlation between PCGs and radioresponse was analyzed using weighted gene co-expression network analysis (WGCNA). The three online databases were used to predict the potential target miRNAs of lncRNAs and the miRNAs that might regulate PCGs. RT-qPCR was utilized to detect the expression of lncRNAs and PCGs in our PCa patients. A total of 65 differentially expressed lncRNAs and 468 differentially expressed PCGs were found between the two groups of PCa. After the chi-square test, LINC01600 was selected to be highly correlated with radioresponse from the 65 differentially expressed lncRNAs. Pearson correlation analysis found 558 PCGs co-expressed with LINC01600. WGCNA identified the darkred module associated with radioresponse in PCa. After taking the intersection of the darkred module of WGCNA, differentially expressed PCGs between the two groups of PCa, and the PCGs co-expressed with LINC01600, three PCGs, that is, JUND, ZFP36, and ATF3 were identified as the potential target PCGs of LINC01600. More importantly, we detected the expression of LINC01600 and three PCGs using our PCa patients, and finally verified that LINC01600 and JUND were differentially expressed between CR and non-CR groups, excluding ZFP36 and ATF3. Meantime, the potential regulation ability of LINC01600 for JUND in PCa cell lines was initially explored. In addition, we constructed the competing endogenous RNA (ceRNA) network of LINC01600—miRNA—JUND. In conclusion, we initially reveal the association of LINC01600 with radioresponse in PCa and identify its potential target PCGs for further basic and clinical research.

## Introduction

Prostate cancer (PCa) is one of the common malignant tumors and the second leading cause of cancer-related death in men (1, 2). Radiotherapy is considered as a standard treatment for primary and advanced PCa. However, 20–40% of high-risk PCa patients experience tumor recurrence or distant metastases with one of the causes is radioresistance (3–5). It has been reported that radioresistance cell reduces the production of reactive oxygen species [ROS; (6)], increases the activation of DNA repair (7), and demonstrates high migratory and invasive abilities (8). However, there are no effective biomarkers to predict radioresponse in PCa patients and the molecular mechanisms underlying radioresistance in PCa have not been clearly elucidated. Thus, it is necessary to find novel biomarkers for predicting radioresponse of PCa patients and elucidate the biological mechanisms of radioresistance of PCa in order to provide new strategies for the personalized treatment of PCa patients.

Long non-coding RNAs (lncRNAs) are a group of RNAs longer than 200 nucleotides, which have many structural features of the mRNAs (9, 10). Current studies indicate that lncRNAs are involved in proliferation, apoptosis, migration, and metastasis (11, 12). Increasing evidence has revealed that lncRNAs are implicated in PCa radioresistance (13), metastatic progression (14), and prognosis (15). However, most previous studies focus on a single lncRNA based on small sample sizes (16–18). Therefore, it is greatly important to explore the correlation between lncRNAs and PCa radioresponse based on large sample high-throughput transcriptome sequencing data.

This study screened the lncRNAs associated with radioresponse in PCa by The Cancer Genome Atlas (TCGA), which is a large comprehensive database including clinical data and transcriptome sequencing data. Differential expression analysis and chi-square test were used to identify lncRNAs associated with radioresponse. Pearson correlation analysis, differential expression analysis, and weighted gene co-expression network analysis (WGCNA) were used to identify the potential target protein-coding genes (PCGs) of lncRNAs. WGCNA is a system biology method that is increasingly used in molecular oncology to cluster highly related genes into modules (19, 20). It is successfully used to explore the association between gene expression and clinical characteristics and to identify candidate biomarkers (21). More importantly, we verified the differential expression of LINC01600 and JUND between complete response (CR) group and non-complete response (non-CR) group using our collected PCa patients, and initially explored the potential regulation ability of LINC01600 for JUND in PCa cell lines. In addition, we constructed a competing endogenous RNA (ceRNA) network of lncRNA—microRNA (miRNA)—PCG using three online databases.

To our knowledge, this study is the first to screen lncRNAs associated with radioresponse in PCa by large sample high-throughput transcriptome sequencing data. We initially reveal the association of LINC01600 with radioresponse in PCa and identify its potential target PCGs for further basic and clinical research.

## Materials and Methods

### Patients Selection, lncRNAs and PCGs Screening

The transcriptome sequencing data and clinical data were collected from TCGA database (https://cancergenome.nih.gov/). LncRNAs and PCGs were isolated from transcriptome sequencing data. A total of 500 PCa patients were found in TCGA database. We selected 31 PCa patients with complete data for this study. Based on the radioresponse, 31 patients were categorized into CR group (*n* = 15) and non-CR group (*n* = 16) which contained partial response, stable disease, and progressive disease. Their clinical characteristics were shown in Supplementary Table 1.

With normalized data, the differential expression of lncRNAs and PCGs between CR group and non-CR group was analyzed using the edgeR package in R software. The fold change (FC) and false discovery rate (FDR) for each lncRNA and PCG were calculated. The cut-off criteria was logFC > 1 and FDR < 0.05.

To validate the bioinformatics analysis results, we further collected the tumor tissues and clinical characteristics of 40 PCa patients who received radiotherapy from the First Affiliated Hospital, Anhui Medical University, which were categorized into CR group (*n* = 20) and non-CR group (*n* = 20) according to their radioresponse. Their clinical characteristics were shown in Supplementary Table 1. All of human studies in our study were in accordance with Declaration of Helsinki and the guidelines of the Committee for Ethical Review of Research at the First Affiliated Hospital, Anhui Medical University.

### Transcriptome Sequencing Data Processing

PCG-seq data was isolated from transcriptome sequencing data. A total of 17,964 PCGs were identified for each sample. The variance analysis was performed, and it was ranked from large to small. The top 25% of the PCGs (4,491 PCGs) with larger variance were selected for WGCNA analysis.

### Weighted Gene Co-expression Network Construction

The expression profile of these 4,491 PCGs was constructed to a gene co-expression network using the WGCNA package in R software (21). The weighted co-expression relationship among all dataset subjects in an adjacency matrix was assessed by the Pearson correlation. In this study, the soft threshold was set as β = 9 (scale-free *R*^2^ = 0.93) to ensure a scale-free network. The adjacency matrix was used to calculate the topological overlap measurement (TOM) representing the overlap in the shared neighbors to further identify functional modules in the co-expression network with these 4491 PCGs (22).

### Identification of Clinical Significant Modules

We performed principal component analysis (PCA) of all PCGs in each module, and defined the value of principal component one as module eigengenes (MEs). MEs were deemed to be representative of gene expression profiles in the module. Next, we performed a correlation analysis between the MEs of each PCG module and the clinical characteristics of PCa patients to identify the clinical significant module.

### Function and Pathway Enrichment Analysis

Gene Ontology (GO) and Kyoto Encyclopedia of Genes and Genomes (KEGG) pathway enrichment analysis of PCGs co-expressed with LINC01600 were performed using Database for Annotation, Visualization, and Integrated Discovery (DAVID) [(23); version 6.8; https://david.ncifcrf.gov/] online functional annotation tool. GO analysis includes three categories: biological processes (BP), cellular components (CC), and molecular functions (MF). Enriched GO terms and KEGG pathways were determined based on the cut-off criteria of adjusted *P* < 0.05.

### Cells, Reagents, and Transfection

The human PCa cell lines including PC-3 and DU145 were purchased from Shanghai Institutes for Biological Sciences, Chinese Academy of Sciences (Shanghai, China), and all cells were cultured in Dulbecco's Modified Eagle's Medium (DMEM) supplemented with 10% fetal bovine serum (FBS) at 37 °C with 5% CO_2_ in a humidified incubator. Small interfering RNA (siRNA) for LINC01600 was designed and synthesized by GenePharma (Shanghai, China). The si-LINC01600 sequence was shown in Supplementary Table 2. Cells were cultured in six-well-plates until 70% confluent, and then transfected with siRNA using Lipofectamine 2000 (Invitrogen) according to the manufacturer's protocol. After incubating the cells for 48 h at 37°C in a humidified chamber supplemented with 5% CO_2_, the cells were harvested for RT-qPCR.

### Reverse Transcription-Quantitative Polymerase Chain Reaction (RT-qPCR)

Total RNA was isolated from PCa tissues using TRIzol reagent (Invitrogen, California, USA). The cDNA was synthesized using HiScript III RT SuperMix for qPCR Kit (Vazyme Biotech, Nanjing, China), according to the manufacturer's protocol at 37°C for 15 min and 85°C for 5 s. The cDNA was subsequently analyzed using ChamQ Universal SYBR qPCR Master Mix (Vazyme Biotech, Nanjing, China) and the ABI7500 system (Applied Biosystems, Foster City, USA). The amplification program was as follows: Initial denaturation step at 95°C for 30 s, followed by 40 cycles at 95°C for 10 s, 60°C for 30 s. The expression of LINC01600, JUND, ZFP36, and ATF3 was calculated relative to the internal reference gene, GAPDH, using the 2^−ΔΔ*Ct*^ method (24). The primer sequences were shown in Supplementary Table 2.

### Competing Endogenous RNA Network Construction

The potential target miRNAs of lncRNAs were predicted by miRcode (25) (http://www.mircode.org/) online database. The miRNAs that might regulate PCGs were predicted by TargetScan (26) (http://www.targetscan.org/vert_72/) and DIANA [(27) diana.imis.athena-innovation.gr/DianaTools/index.php] online databases. Venn diagram was used to confirm overlapping miRNAs. Cytoscape software (version 3.6.1; https://cytoscape.org/) was used to visual the ceRNA network.

### Statistical Analysis

Statistical analysis was constructed by SPSS (version 24.0) and R software (version 3.4.2; https://www.r-project.org/). Pearson correlation analysis was used to identify the PCGs co-expressed with lncRNAs. The chi-square test was used to identify the correlation between lncRNAs and radioresponse. *P* < 0.05 was considered statistically significant.

## Results

### Differentially Expressed lncRNAs and PCGs

According to the cut-off criteria (logFC > 1, FDR < 0.05), 65 differentially expressed lncRNAs (Supplementary Table 3) and 468 differentially expressed PCGs (Supplementary Table 4) were identified between CR group and non-CR group. LINC01600 (logFC = 1.0046, FDR = 0.0155) and JUND (logFC = 1.0210, FDR = 0.0002) were upregulated in non-CR group.

### Identification of lncRNAs Associated With Radioresponse

To further identify lncRNAs which were highly correlated with radioresponse from 65 differentially expressed lncRNAs, we performed the chi-square test. According to the median value, the expression of lncRNAs was divided into low and high expression. LINC00919 and LINC02010 were excluded because more than half of the cases did not detect their expression. LINC01600 (*P* = 0.004), LINC01060 (*P* = 0.012), MIR137HG (*P* = 0.032), LINC02006 (*P* = 0.032), and LINC02531 (*P* = 0.032) were identified as highly correlated with radioresponse (Table 1 and Supplementary Table 5). We chose LINC01600 with the lowest *P*-value for subsequent analysis.

**Table 1:** Chi-square test between lncRNA expression and radioresponse.

**lncRNA**	**Expression level**	**CR group**	**non-CR group**	***P*-value**
LINC01600	Low	12	4	**0.004**
	High	3	12	
LINC02006	Low	11	5	**0.032**
	High	4	11	
LINC02531	Low	11	5	**0.032**
	High	4	11	
MIR137HG	Low	11	5	**0.032**
	High	4	11	
LINC01060	Low	4	12	**0.012**
	High	11	4	

*CR, complete response; non-CR, non-complete response. The P-value indicating statistical significance is marked with bold type*.

### Weighted Gene Co-expression Network Construction and Key Modules Identification

**Figure 1:**
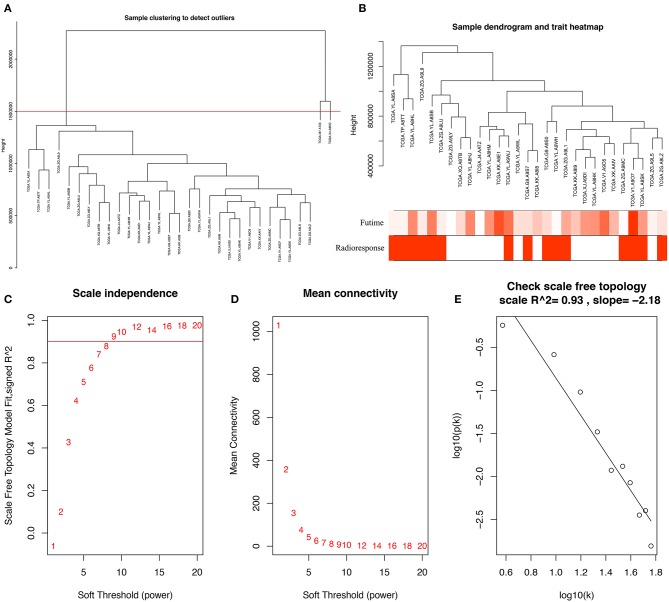
**(A)** Clustering dendrogram of 31 samples, and excluding two outlier sample. **(B)** Clustering dendrogram of 29 samples corresponding to clinical characteristics. **(C)** Analysis of the scale-free fit index for various soft-thresholding powers (β). **(D)** Analysis of the mean connectivity for various soft-thresholding powers. **(E)** Checking the scale-free topology when β = 9.

To identify PCGs associated with radioresponse in PCa, we performed a WGCNA analysis. The samples of 31 PCa patients receiving radiotherapy were clustered using average linkage method and Pearson correlation analysis. We excluded 2 outlier samples and finally included 29 samples for subsequent analysis (Figures 1A,B). Next, we performed a network topology analysis of various soft-thresholding powers to have relatively balanced scale independence and average connectivity of WGCNA. In our study, the power of β = 9 (scale free *R*^2^ = 0.93) was selected as the soft-thresholding parameter to make sure a scale-free network (Figures 1C–E).

**Figure 2:**
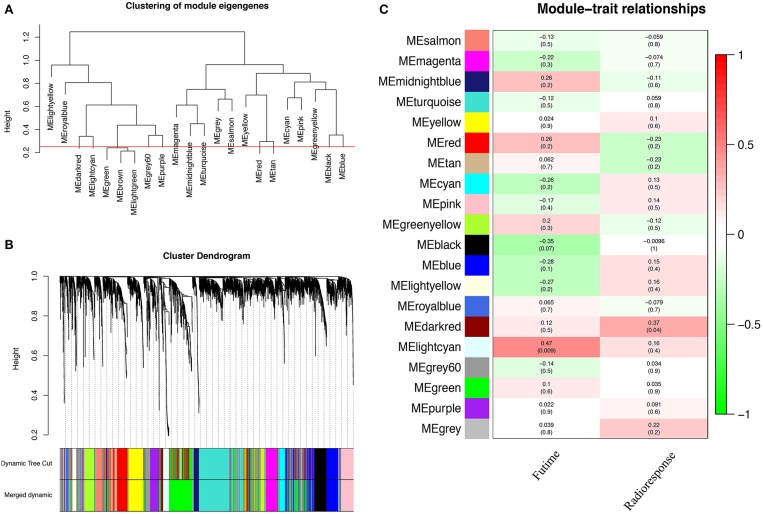
Identification of PCG modules associated with the radioresponse of PCa. **(A)** The horizontal line defines the threshold, so 20 distinct PCG modules are identified. **(B)** The dendrogram of all genes is clustered based on a dissimilarity measure (1-TOM). **(C)** The heatmap shows the correlation between MEs and the radioresponse of PCa. Red represents a positive correlation between PCG modules and clinical characteristics, and green represents a negative correlation between PCG modules and clinical characteristics.

**Figure 3:**
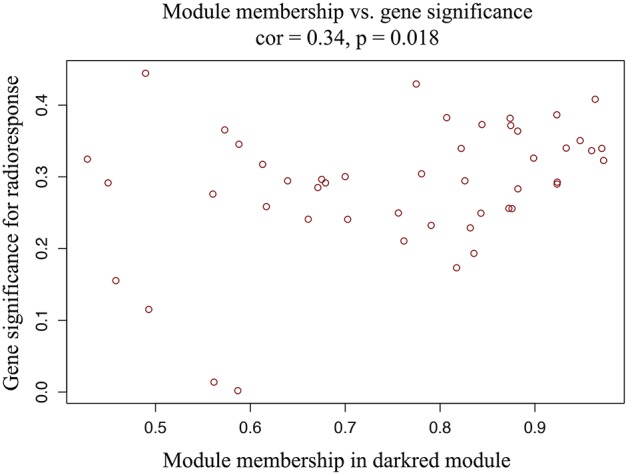
The scatter plot shows the correlation between gene significance for radioresponse and module membership in darkred module (Cor = 0.34, *P* = 0.018).

A total of 22 PCG modules were generated in a hierarchical clustering tree through dynamic tree cut and merged dynamics. After merging modules with dissimilarity less than 25%, 20 distinct PCG modules were identified (Figures 2A,B). Correlation analysis was performed between the MEs of each PCG module and radiotherapy outcome. According to *P* < 0.05 of correlation analysis, the darkred module (Cor = 0.37, *P* = 0.04) was identified as the PCG module highly correlated with radioresponse (Figure 2C). Subsequently, we calculated the gene significance for radioresponse and the module membership in darkred module. The scatter plot (Cor = 0.34, *P* = 0.018) further validated the high correlation between the darkred module and radioresponse (Figure 3). The list of PCGs for the darkred module (*n* = 48) was shown in Supplementary Table 6.

### Identifying the Potential Target PCGs for LINC01600

In order to screen the potential target PCGs for LINC01600, we performed Pearson correlation analysis between LINC01600 and 17964 PCGs. According to the cut-off criteria of | Pearson correlation coefficient | > 0.4 and *P* < 0.05, a total of 558 PCGs co-expressed with LINC01600 were screened (Supplementary Table 7).

**Figure 4:**
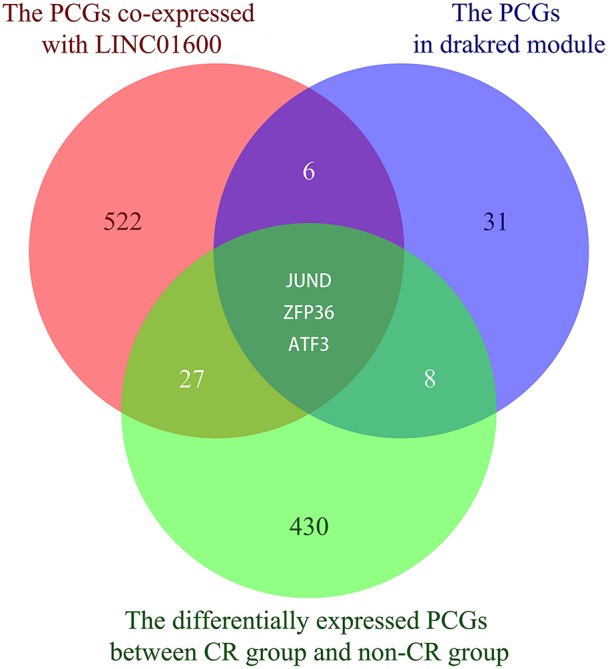
The Venn diagram shows the intersection of the 558 PCGs co-expressed with LINC01600, the 48 PCGs in the drakred module obtained by WGCNA, and the 468 differentially expressed PCGs between CR group and non-CR group.

After taking the intersection of the 558 PCGs co-expressed with LINC01600, the 48 PCGs in the drakred module obtained by WGCNA, and the 468 differentially expressed PCGs between CR group and non-CR group, we obtained the potential target PCGs of LINC01600, that is, JUND, ZFP36, and ATF3 (Figure 4).

### GO and KEGG Pathway Enrichment Analysis

**Figure 5:**
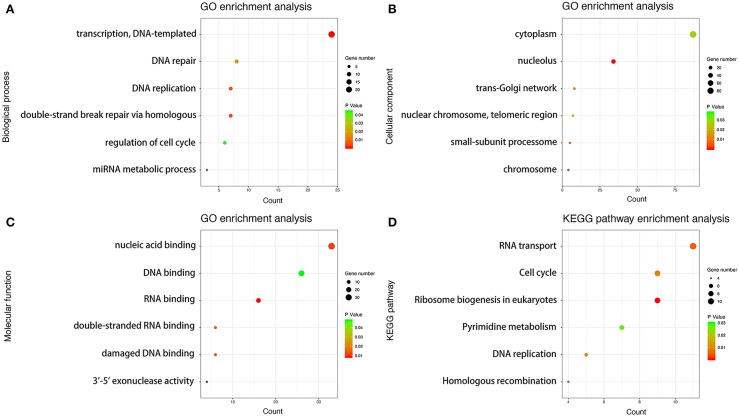
GO and KEGG pathway enrichment analysis. **(A)** Biological process analysis. **(B)** Cellular component analysis. **(C)** Molecular function analysis. **(D)** KEGG pathway analysis.

We performed GO enrichment analysis on 558 PCGs co-expressed with LINC01600. The PCGs in BP group were mainly enriched in transcription, regulation of cell cycle, miRNA metabolic process, double-stand break repair via homologous recombination, DNA replication, and DNA repair. The PCGs in CC group were mainly enriched in trans-Golgi network, small subunit processome, nucleolus, nuclear chromosome, telemetric region, cytoplasm, and chromosome. The PCGs in MF group were mainly enriched in RNA binding, nucleic acid binding, double-stranded RNA binding, DNA binding, damaged DNA binding, and 3–5 exonuclease activity. KEGG pathway analysis revealed that these PCGs were mainly involved in RNA transport, ribosome biogenesis in eukaryotes, pyrimidine metabolism, homologous recombination, DNA replication, and cell cycle (Figure 5). These results indicated that the 558 PCGs co-expressed with LINC01600 were involved in various biological processes such as DNA damage repair, metabolism, cell cycle, and so on, which might be related to cell radioresponse.

### Verifying the Differential Expression of LINC01600 and JUND

**Figure 6:**
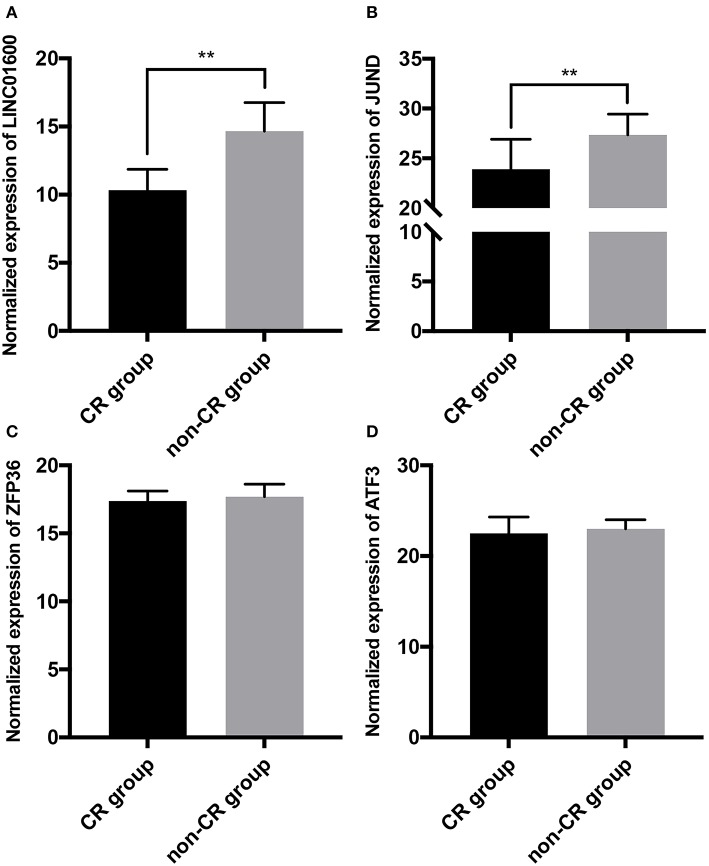
The expression of **(A)** LINC01600, **(B)** JUND, **(C)** ZFP36, and **(D)** ATF3 is detected by RT-qPCR in tumor tissues of 40 PCa patients collected in our hospital. LINC01600 and JUND are significantly upregulated in non-CR group compared with CR group. There is no differential expression of ZFP36 and ATF3 between CR group and non-CR group (**P < 0.01).

RT-qPCR was used to detect the expression of LINC01600, JUND, ZFP36, and ATF3 in tumor tissues of 40 PCa patients collected in our hospital. The radioresponse of 40 PCa patients receiving radiotherapy included 20 CR and 20 non-CR. We found that LINC01600 and JUND were significantly upregulated in non-CR group compared with CR group (Figures 6A,B). However, there was no differential expression of ZFP36 and ATF3 between CR group and non-CR group (Figures 6C,D). These results initially verified that LINC01600 and JUND were associated with radioresponse in PCa. We chose JUND as the potential target PCGs of LINC01600 for subsequent analysis.

### Preliminary Validation of the Target PCG of LINC01600

**Figure 7:**
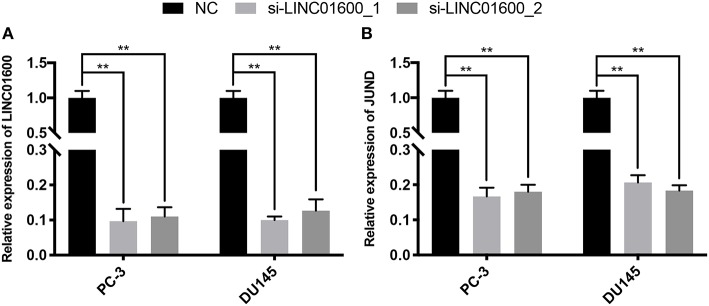
The expression of LINC01600 and JUND in PC-3 and DU145 cell lines is detected by RT-qPCR. After downregulating **(A)** the expression of LINC01600 by si-LINC01600_1 and si-LINC01600_2, respectively and **(B)** the mRNA expression of JUND is also downregulated (**P < 0.01).

LINC01600 was downregulated by siRNA at two different sites in PCa cell line. The lncRNA expression of LINC01600 and the mRNA expression of JUND were detected by RT-qPCR. The results showed that after downregulating LINC01600 by si-LINC01600_1 and si-LINC01600_2, the mRNA expression of JUND was also downregulated (Figure 7). In summary, LINC01600 may regulate JUND.

### The Network of ceRNA Construction

**Figure 8:**
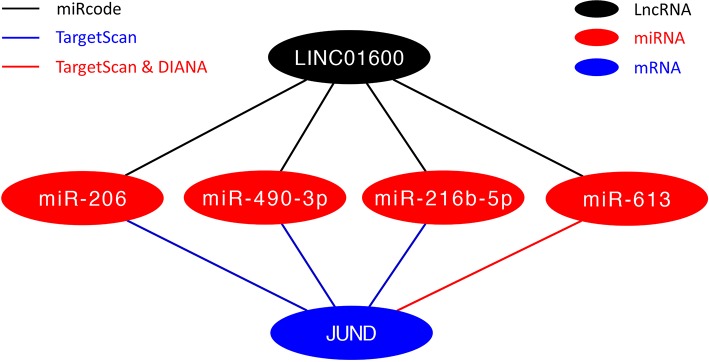
The ceRNA network of LINC01600—miRNAs—JUND.

To further explore the potential mechanisms of LINC01600, we constructed a ceRNA network of LINC01600—miRNAs—JUND. The miRcode database was used to predict the potential target miRNAs of LINC01600. The TargetScan and DIANA databases were used to predict the miRNAs that might regulate JUND. After taking the intersection of the above two results, four miRNAs, that is, miR-206, miR-490-3p, miR-216b-5p, and miR-613, were screened for constructing the ceRNA network (Figure 8).

## Discussion

In recent years, the incidence of PCa is significantly increased, although radiotherapy has achieved great success in the treatment of advanced PCa, ~10–45% of PCa is resistant to irradiation. Therefore, it is extremely important to find novel biomarkers that can predict radioresponse, so as to find new therapeutic targets and develop new modalities of the treatment. Increasing evidence has indicated that lncRNAs play important roles in cancer pathologies, prognosis, and therapeutic options. Several previous studies have illustrated that some lncRNAs associated with radiosensitivity in PCa, which are GAS5 (17), UCA1 (28), and HULC (13). However, the above three studies examine the expression of the lncRNAs by RT-qPCR in different PCa cell lines only. The relationship between the expression of lncRNAs in PCa patients and radioresponse is still unclear. To our knowledge, this study is the first to employ large sample high-throughput transcriptome sequencing data and clinical data from TCGA database to screen lncRNA associated with radioresponse in PCa and to explore its potential biological mechanisms.

Through the analysis of the TCGA datasets, a total of 65 differentially expressed lncRNAs and 468 differentially expressed PCGs were found between CR group and non-CR group in radioresponse of PCa. LINC01600 (logFC = 1.0046, FDR = 0.0155) and JUND (logFC = 1.0210, FDR = 0.0002) were upregulated in non-CR group compared with CR group. After chi-square test, five lncRNAs were selected to be highly correlated with radioresponse from the 65 differentially expressed lncRNAs. We chose LINC01600 with the lowest *P*-value for subsequent analysis. The previous study indicates that LINC01600 is upregulated in lung adenocarcinoma (LUAD) and its promoter and enhancer show increased activity in luciferase reporter assays. The patients with high expression of LINC01600 in LUAD have poor prognoses (29). This indicates that LINC01600 is a carcinogenic lncRNA.

We further explore the potential target PCGs of LINC01600. Pearson correlation analysis found 558 PCGs co-expressed with LINC01600. GO and KEGG enrichment analysis results showed that the 558 PCGs co-expressed with LINC01600 were involved in various biological processes such as DNA damage repair, metabolism, cell cycle, and so on. Many studies have shown that DNA damage repair, metabolism, and cell cycle are highly correlated with radiosensitivity (30–34). This further indicates that LINC01600 and the PCGs co-expressed with it are associated with radioresponse. WGCNA identified the darkred module associated with radioresponse in PCa. After taking the intersection of the 558 PCGs co-expressed with LINC01600, the 48 PCGs in the drakred module obtained by WGCNA, and the 468 differentially expressed PCGs between CR group and non-CR group, 3 PCGs were identified as the potential target PCGs of LINC01600. More importantly, the RT-qPCR results of our collected PCa patients showed that LINC01600 and JUND were highly expressed in non-CR group compared with CR group in radioresponse. After downregulating LINC01600 in PCa cell lines, the mRNA expression of JUND was also downregulated, which indicates that LINC01600 may regulate JUND.

Studies have shown that the expression of JUND is positively correlated with tumor cell proliferation in diffuse large B-cell lymphomas (35). JUND accelerates tumor cell growth, inhibits apoptosis and enhances invasion in non-small cell lung cancer (36). Selective knockdown of JUND expression using siRNA in DU145 and PC3 cells results in significant reduction in cell proliferation, and forced overexpression of JUND increases the proliferation rate in PCa (37). Meanwhile, JUND contributes to escape from programmed cell death and confer radio-resistance of prostate cancer (PCa) cells (38). These results indicate that JUND is an oncogene and it enhances the proliferation capacity and confers radio-resistance of PCa cells. In our study, LINC01600 is identified as an upstream regulator of JUND, which may be involved in the regulation of radioresponse in PCa.

To further explore the potential biological mechanisms of LINC01600, we constructed a ceRNA network of LINC01600—miRNAs—JUND using three online databases, that is, miRcode, TargetScan, and DIANA. After taking the intersection of the potential target miRNAs of LINC01600 and the miRNAs that might regulate JUND, 4 miRNAs, that is, miR-206, miR-490-3p, miR-216b-5p, and miR-613, were screened to construct the ceRNA network. Studies have shown that miR-206 inhibits proliferation and migration of PCa cells by targeting CXCL11 (39). MiR-490-3p inhibits the growth and invasiveness in triple-negative breast cancer by repressing the expression of TNKS2 (40). Overexpression of LINC00518 contributes to the paclitaxel resistance in PCa via sequestering miR-216b-5p (41). MiR-613 inhibits bladder cancer proliferation and migration through targeting SphK1 (42). These results indicate that miR-206, miR-490-3p, miR-216b-5p, and miR-613 are tumor suppressors.

In summary, our study initially reveals the association of LINC01600 with radioresponse in PCa and explores its potential biological mechanisms for further basic and clinical research.

## Data Availability Statement

The datasets for this study can be found in the TCGA database https://cancergenome.nih.gov/.

## Ethics Statement

This study was approved by the ethics committee of the First Affiliated Hospital of Anhui Medical University. All subjects gave written informed consent in accordance with the Declaration of Helsinki.

## Author Contributions

MX is responsible for research design, data collection, statistical analysis, RT-qPCR experiments, and manuscript writing. SG and YL are responsible for research design, data collection, and bioinformatics analysis. JZ, JD, and CY are responsible for providing the PCa specimens. MY and FZ are responsible for data collection. CL and ZT guide research ideas, design, research methods, and manuscript revision.

### Conflict of Interest

The authors declare that the research was conducted in the absence of any commercial or financial relationships that could be construed as a potential conflict of interest.
