# Supplementary material for: Identifying Long Non-coding RNA of Prostate Cancer Associated With Radioresponse by Comprehensive Bioinformatics Analysis
Source: Front Oncol. 2020 Apr 7;10:498. doi: 10.3389/fonc.2020.00498 (PMC7154134; doi:10.3389/fonc.2020.00498)
Supplement: Supplementary file 1 [file Table_1.DOCX]

**Supplementary Table 1.** The clinical characteristics of 31 PCa patients receiving radiotherapy in TCGA database.

| Variables | CR group | non-CR group |
| --- | --- | --- |
| Age at initial diagnosis | |  |
| <65 | 6 | 8 |
| >=65 | 9 | 8 |
| T stage |  |  |
| T3a | 3 | 5 |
| T3b | 11 | 11 |
| T4 | 1 | 0 |
| N stage |  |  |
| N0 | 8 | 8 |
| N1 | 7 | 8 |
| M stage |  |  |
| M0 | 15 | 16 |

CR = complete response, non-CR = non-complete response.

**Supplementary Table 1.** The clinical characteristics of 40 PCa patients receiving radiotherapy in our hospital.

| Variables | CR group | non-CR group |
| --- | --- | --- |
| Age at initial diagnosis | |  |
| <65 | 7 | 9 |
| >=65 | 13 | 11 |
| T stage |  |  |
| T2a | 1 | 0 |
| T2b | 0 | 1 |
| T2c | 1 | 2 |
| T3a | 5 | 5 |
| T3b | 12 | 10 |
| T4 | 1 | 2 |
| N stage |  |  |
| N0 | 10 | 13 |
| N1 | 10 | 7 |
| M stage |  |  |
| M0 | 20 | 20 |

CR = complete response, non-CR = non-complete response.
