# Supplementary material for: Identifying Long Non-coding RNA of Prostate Cancer Associated With Radioresponse by Comprehensive Bioinformatics Analysis
Source: Front Oncol. 2020 Apr 7;10:498. doi: 10.3389/fonc.2020.00498 (PMC7154134; doi:10.3389/fonc.2020.00498)
Supplement: Supplementary file 2 [file Table_2.DOCX]

**Supplementary Table 2.** The siRNA sequences.

| lncRNA | siRNA |
| --- | --- |
| LINC01600_1 | sense, 5'-GCACAUUCAUAAUGGACAUTT-3' |
|  | antisense, 5'-AUGUCCAUUAUGAAUGUGCTT-3' |
| LINC01600_2 | sense, 5'-GCUAAUUUCUCAGCUGUAATT-3' |
|  | antisense, 5'-UUACAGCUGAGAAAUUAGCTT-3' |
| Negative control | sense, 5′-UUCUCCGAACGUGUCACGUTT-3′ |
|  | antisense, 5′-ACGUGACACGUUCGGAGAATT-3′ |

**Supplementary Table 2.** The primer sequences.

| lncRNA and PCG | Primer Sequence |
| --- | --- |
| LINC01600 | forward, 5′-GCTCACCGTAATGCTCACAACAAC-3′ |
|  | reverse, 5′-GTGTCTGAGAGCCGTTGAGTCTTC-3′ |
| JUND | forward, 5′-TCAAGACCCTCAAAAGCCAGA-3′ |
|  | reverse, 5′-CGTGGCTGAGGACTTTCTGTT-3′ |
| ZFP36 | forward, 5′-TTTTACGACGACGGCAACGG-3′ |
|  | reverse, 5′-AGCGGCATCAGGTTGAGGTC-3′ |
| ATF3 | forward, 5′-TTCTCCCAGCGTTAACACAAAA-3′ |
|  | reverse, 5′-AGAGGACCTGCCATCATGCT-3′ |
| GAPDH | forward, 5′-AAGGTGAAGGTCGGAGTCAA-3′ |
|  | reverse, 5′-AATGAAGGGGTCATTGATGG-3′ |

PCG = protein-coding gene.
